# Long-Term Follow-Up and Survivorship After Completing Systematic Surveillance in Stage I–III Colorectal Cancer: Who Is Still at Risk?

**DOI:** 10.1007/s12029-015-9723-2

**Published:** 2015-04-28

**Authors:** T. Veen, K. Stormark, B. S. Nedrebø, M. Berg, J. A. Søreide, H. Kørner, Kjetil Søreide

**Affiliations:** Department of Gastrointestinal Surgery, Stavanger University Hospital, P.O. Box 8100, 4068 Stavanger, Norway; Centre for Organelle Research, Faculty of Science and Technology, University of Stavanger, 4036 Stavanger, Norway; Department of Clinical Medicine, University of Bergen, P.O. Box 7804, 5020 Bergen, Norway

**Keywords:** Colorectal cancer, Survival, Long-term follow-up, Surveillance, Recurrence, Microsatellite instability

## Abstract

**Purpose:**

In patients with a high life expectancy at the time of surgery for colorectal cancer (CRC), the long-term outcome may be influenced by factors other than their cancer. We aimed to investigate the long-term outcome and cause of death beyond a 5-year surveillance programme.

**Methods:**

We evaluated the overall survival (OS) and cancer-specific survival (CSS) of a population-based cohort of stage I–III CRC patients <75 years old who completed a systematic surveillance programme.

**Results:**

In total, 161 patients <75 years old, 111 (69 %) of whom were node negative (pN0), were included. The median follow-up time was 12.1 years. The OS was 54 % at 15 years and differed significantly between the pN0 and pN+ patients (65 vs. 30 %; *P* < 0.001); CSS (72 %) also differed between the pN0 and pN+ patients (85 vs. 44 %; *P* < 0.001). For the 5-year survivors (*n* = 119), 14 (12 %) died of CRC during additional long-term follow-up (7 each for pN0 and pN+), and 6 patients (5 %; all pN0) died of other cancers. Patients aged <65 years exhibited better long-term survival (81 %), but most of the deaths were due to CRC (10/12 deaths). Only two of the 14 cancer-related deaths involved microsatellite instable (MSI) CRC. Females exhibited better OS and CSS beyond 5 years of surveillance.

**Conclusions:**

The long-term survival beyond 5-year survivorship for stage I–III CRC is very good. Nonetheless, cancer-related deaths are encountered in one-third of patients and occur most frequently in patients who are <65 years old at disease onset—pointing to a still persistent risk several years after surgery.

## Introduction

Colorectal cancer (CRC) is a leading cause of cancer-related morbidity and mortality in the Western world. The prognosis for CRC has increased considerably with the development of better staging, improved surgical techniques and adjuvant therapy [1–3]. Indeed, the 5-year survival rates have never been better, and survival in rectal cancer has now even surpassed that of colon cancer [4]. Cancer-specific survival for all colorectal patients now exceeds 60 % when a curative resection is performed [1, 4–8]. Overall, the presence of lymph node metastasis is one of the strongest prognostic factors in CRC, but the appropriate therapy remains a controversial and much-debated topic [9]. Patients with node-negative disease (pN0) have excellent prognoses, but up to 10–20 % of cases may nonetheless recur, develop distant metastasis and eventually die from disseminated disease [10]. Even T1 node-negative disease patients exhibit a 7–8 % risk of cancer-related death at the 10-year follow-up [11].

Systematic surveillance has been suggested to detect asymptomatic recurrences at a curable stage; however, the evidence for this approach and the potential follow-up strategies remain debated [12]. First, the choice of modality for surveillance is unclear, although some form of imaging and monitoring with tumour markers (i.e., carcinoembryonic antigen; CEA) is used. However, this method of surveillance is not currently tailored to the individual patient’s risk for recurrence [13]. Second, the recurrence pattern and mortality risk may differ throughout the course of follow-up, which is demonstrated by the time differences in colon and rectal cancer mortality [14]. Third, patients presenting with cancer at a young age may have a different disease pattern and recurrence risk than elderly patients [15]. Indeed, although the 5-year follow-up is satisfactory in patients who are elderly, with limited long-term longevity, the younger patient population may exhibit an increased lifetime risk for both disease recurrence and death from disease. However, very little data exist on very long-term outcomes for the younger (<75 years old at diagnosis) population of patients with colorectal cancer, and there is even less information concerning patients who are <65 years old. As the expected lifespan is currently increasing (approaching over 80 years of age for both genders in Norway) [16], it is increasingly important to determine the long-term survival of the youngest (e.g. <75 years of age) CRC patients who are curatively treated.

Consequently, the aim of this study was to examine the very long-term (up to 15 years) survival of CRC patients after curative resection, specifically survival beyond 5 years, in a systematic surveillance programme after surgery.

## Methods

The study was approved as a quality assurance project (REK#2010/3414) by the Regional Ethics Committee of the Health Trust of Western Norway. Informed consent was waived because this was a quality assurance of clinical practice.

### Study Cohort and Background

The study cohort included a set of long-term follow-up patients who have been thoroughly described elsewhere [17]. Briefly, all patients with CRC who underwent operations at Stavanger University Hospital between July 1996 and June 1999 were included in this study. Stavanger University Hospital serves as the only hospital for the population in the region, with an estimated 280,000 inhabitants at the time the study commenced.

During the study period, 314 patients had curative treatment, and 194 of these patients were enrolled in a postoperative systematic follow-up programme according to the existing guidelines from Norwegian Gastrointestinal Cancer Group (NGICG) [18]. The follow-up was based on regular CEA-monitoring, chest X-ray, liver ultrasound, and 1- and 5-year colonoscopy [17, 19].

### Inclusion and Exclusion Criteria

All 194 patients who had an R0 resection (defined as free resection borders by microscopy) for colon or rectal cancer and were included in the systematic surveillance programme were eligible. Further, patients with available DNA for microsatellite instability (MSI) analysis were included (*n* = 186, 96 % of the 194), as previously reported [3]. Those patients who were <75 years at the time of primary diagnosis were included in the current study.

### Follow-Up Beyond 5 Years After Curative Surgery

The follow-up was performed by searching the hospital’s electronic and paper records for information. All patients in Norway have an 11-digit social security number linked to several population-based registries, including the Cause of Death registry. The Cause of Death registry is connected to the hospital electronic system, and a registered death (and date of death) of a patient is immediately marked in the electronic hospital files (if death occurs in the hospital) or is marked within a short lag time if the death occurs outside the hospital or in another geographic area. Therefore, we were able to detect all deaths that occurred until an arbitrarily set date 3 weeks before the final follow-up date of July 23, 2011. Patients were followed until the date of the final follow-up or until death from cancer or another disease. Patients who were lost to follow-up (moved out of the country) were censored with last known hospital contact as the final date.

### Descriptive Data and Characteristics

Cancers were histopathologically staged according to the tumour-node-metastasis (TNM) classification system (5th revision) at the time. Location in the colorectum was defined as ‘proximal’ or ‘distal’; proximal cancers included cancers located from the caecum to the left flexure, and distal cancers were located from the descending colon to the rectum. Pre- and postoperative CEA values were registered and obtained by routine laboratory workup before and after surgery, as previously reported [19, 20]. The preoperative ASA score was recorded. All patients had their MSI status analysed using a PCR-based technique, which was determined according to standard criteria, as previously described [19].

### Study Endpoint

The primary endpoint of the current investigation was cancer-specific long-term survival more than 5 years after completing a systematic surveillance programme as well as overall survival (defined as death from any cause). Additionally, we wanted to record any type of fatal event to compare the overall survival (e.g. from CRC, a new other malignancy, or any other causes) for the youngest group (those <65 years of age at the primary diagnosis) with the oldest group (≥65 years but <75 years old at the primary diagnosis). For cancer-specific survival, the non-cancer-related deaths were censored in the survival analysis.

### Statistical Analyses

Statistical analyses were performed using the IBM Statistical Package for Social Sciences, version 21 (SPSS v. 21). Descriptive data are presented as the median and interquartile range (IQR) for continuous variables, or when appropriate, dichotomised. Survival analyses were performed using the Kaplan-Meier method and log rank test. All tests were two-tailed, and *P* values <0.050 were considered statistically significant.

## Results

### Overall Outcome for Stage I–III Patients

**Fig. 1:**
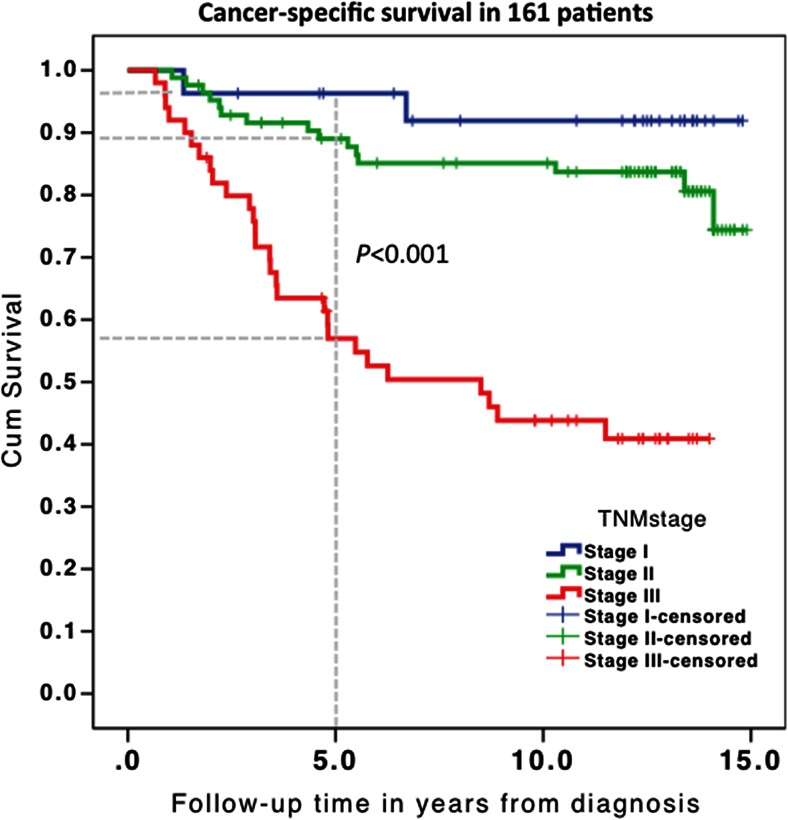
Stage-dependant, cancer-specific, long-term survival for all patients who were <75 years old at diagnosis. Stage-dependant survival is depicted. The *grey dotted vertical line* parallel to the *x*-axis denotes the 5-year point at which systematic surveillance after surgery ended. The corresponding *grey dotted horizontal lines* on the *y*-axis indicate the corresponding stage-dependant survival at 5 years

In total, 161 patients met the inclusion criteria and were ≤75 years old at diagnosis; their clinical characteristics are presented in Table 1. The median follow-up was 12.1 years (interquartile range 4.8–13.4 years). The 5-year overall survival was 72 % (n = 116), and the 5-year cancer-specific survival was 88 % (*n* = 123) for all stages (Table 2). The long-term (up to 15 years) overall survival was 54 % (*n* = 89), which differed significantly between stage I/II (63 and 66 %) and stage III (30 %; *P* < 0.001). The cancer-specific survival for all stages is depicted in Fig. 1 and Table 2.

**Table 1:** Study characteristics of all patients and long-term survivors

Descriptive	All patients, *n* = 161 (%)	Long-term survivors, *n* = 119
Age (median, IQR; years)	64.5 (57.9–69.9)	63.7 (57.6–69.9)
<65 years	83 (52 %)	62 (52 %)
≥65 to 75 years	78 (48 %)	57 (48 %)
Gender		
Male	98 (61 %)	73 (61 %)
Female	63 (39 %)	46 (39 %)
ASA score^a^		
I	92 (57 %)	71 (61 %)
II	57 (35 %)	41 (35 %)
III	10 (6 %)	5 (4 %)
> II	0	0
TNM stage		
I	27 (17 %)	23 (19 %)
II	84 (52 %)	70 (59 %)
III	50 (31 %)	26 (22 %)
Location		
Colon	103 (54 %)	73 (61 %)
Rectum	58 (36 %)	46 (39 %)
Grade		
High/moderate	140 (87 %)	108 (91 %)
Low/mucinous	21 (13 %)	11 (9 %)
T stage		
T1–2	33 (20.5 %)	27 (23 %)
T3–4	128 (79.5 %)	92 (77 %)
MSI status		
MSI	36 (22 %)	25 (21 %)
MSS	125 (78 %)	94 (79 %)
CEA pre-op^b^	3.0 (1.0–6.0)	3.0 (1.0–7.0)
≤4	94 (67 %)	71 (66 %)
>4	47 (33 %)	36 (34 %)
CEA post-op^b^	1.0 (1.0–2.0)	1.0 (1.0–2.0)
≤4	131 (89 %)	99 (88 %)
>4	17 (12 %)	13 (12 %)

Data are presented as the median with interquartile ranges or numbers with rates (%). Percentages may not add up due to rounding

*ASA* American Society of Anesthesiology fitness class, *MSI* microsatellite instability, *CEA* carcinoembryonic antigen

^a^Data missing from two patients

^b^Based on patients with available data

**Table 2:** Cancer-specific survival in long-term survivors (*n* = 119)

	Variable	Total (*n*)	Alive or censored % (*n*)	Dead from CRC % (*n*)	*P* value
Age	<65 years	62	80.6 % (50)	16.1 % (10)	0.197
	≥65 years	57	63.2 % (36)	7.0 % (4)	
Gender	Male	73	65.8 % (48)	16.4 % (12)	0.041
	Female	46	82.6 % (38)	4.3 % (2)	
TNM stage	I + II	93	77.4 % (72)	7.5 % (7)	0.002
	III	26	53.8 % (14)	26.9 % (7)	
Location	Distal	83	71.1 % (59)	10.8 % (9)	0.664
	Proximal	36	75.0 % (27)	13.9 % (5)	
Location	Colon	73	79.5 % (58)	8.2 % (6)	0.113
	Rectum	46	60.9 % (28)	17.4 % (8)	
MSI status	MSI	25	84.0 % (21)	8.0 % (2)	0.483
	MSS	94	69.1 % (65)	12.8 % (12)	
Grade	High/moderate	108	70.4 % (76)	13.0 % (14)	0.233
	Low/mucinous	11	90.9 % (10)	0	
T stage	1 + 2	27	70.4 % (19)	11.1 % (3)	0.892
	3 + 4	92	72.8 % (67)	12.0 % (11)

### Long-Term Survivors After 5-Year Surveillance

In total, 119 patients (74 %) survived beyond 5 years after diagnosis and were therefore subject to long-term analyses. For this sub-cohort, the median age was 63.7 years (interquartile range 57.6–70.0) with a similar distribution of younger (<65 years) and older (>65 to 75 years) patients (52 and 48 %, respectively) (Table 3). Males accounted for 61 % (73/119) of the patients, and the majority of patients had node-negative disease at diagnosis. Twenty-three (19.3 %) patients had stage I disease, 70 (58.8 %) patients had stage II disease and 26 (21.8 %) patients had stage III disease.

**Table 3:** Outcomes after the 5-year follow-up according to age at diagnosis

		Age groups		Median follow-up	Total (*n*)
		<65 years	65 to 75 years		
Follow-up status	Alive^a^	50 (81 %)	36 (63 %)	13.2 years	86 (72 %)
	Dead from CRC	10 (16 %)	4 (7 %)	7.6 years	14 (12 %)
	Dead from another cancer	1 (2 %)	5 (9 %)	9.3 years	6 (5 %)
	Dead from another cause	1 (2 %)	12 (21 %)	10.1 years	13 (11 %)
Total		62 (52 %)	57 (48 %)		119

^a^Alive at the end of follow-up; not censored for death from CRC, another cancer or another cause. The percentages may not add up due to rounding

### Cause of Death in Long-Term Survivors After 5 Years of Surveillance

In total, 33 of the 119 patients died during the follow-up period after 5 years of surveillance. CRC was the cause of death in 14 patients, whereas 13 and 6 patients died of other causes (e.g., heart disease) and other cancers, respectively. Ten of the 14 patients who eventually died of CRC had developed recurrence within 5 years of surveillance. For those who had not died of CRC at the end of the long-term follow-up, a further 9 had developed recurrence, 7 of whom were alive and 2 of whom had died from other causes. Lung cancer, lymphoma and pancreatic cancer were the other cancers that resulted in death, and 2 patients died of a cancer of unknown origin. For the 13 patients who died of other causes, 6 patients died of cardiovascular disease.

### Long-Term Overall and Cancer-Specific Survival

**Fig. 2:**
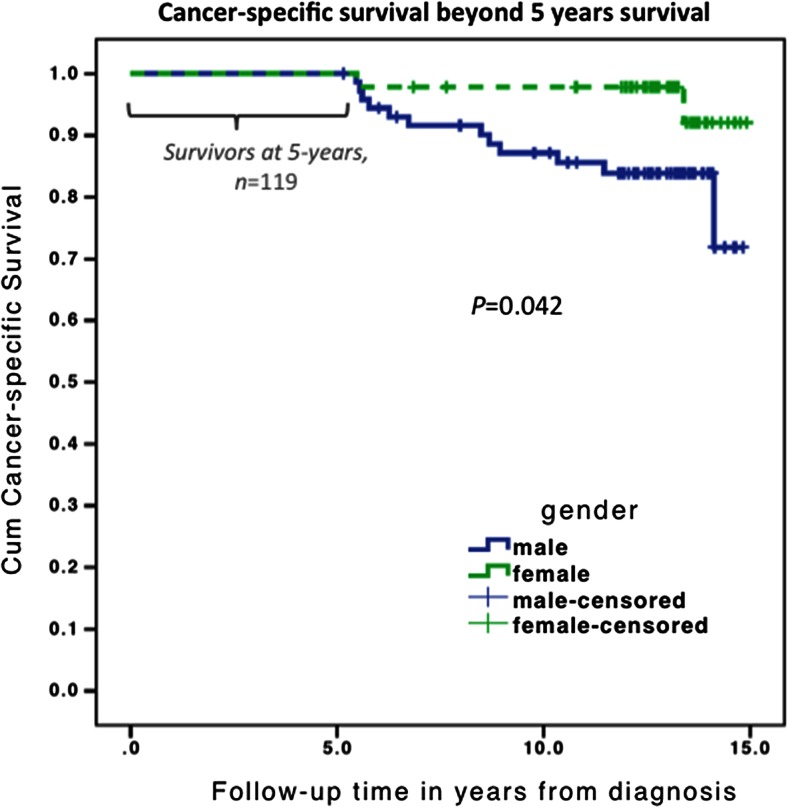
Cancer-specific, long-term survival for patients alive at the 5-year follow-up, stratified for men and women. The first 5 years indicate survival and completed surveillance after surgery. After the 5-year surveillance programme, gender-specific and cancer-specific survival is depicted

For the long-term survivors, overall survival at 15 years was 72 %, and the difference was significant between stages I/II (74 and 79 %, respectively) and III (54 %; *P* = 0.024). When comparing node-negative and node-positive disease, survival at 15 years was 77 vs. 54 % (*P* = 0.007) (Table 4). Univariate cancer-specific survival beyond 5 years of surveillance is presented in Table 2. Only gender (Fig. 2) and TNM stage were significant predictors of survival.

**Table 4:** Outcomes after the 5-year follow-up according to node status

		Node status		Total
		Node negative	Node positive	
Follow-up status	Alive^a^	72 (77 %)	14 (54 %)	86 (72 %)
	Dead from CRC	7 (8 %)	7 (27 %)	14 (12 %)
	Dead from another cancer	6 (7 %)	0	6 (5 %)
	Dead from another cause	8 (9 %)	5 (19 %)	13 (11 %)
Total		93 (78 %)	26 (22 %)	119

The percentages may not add up due to rounding

^a^Alive at the end of follow-up; not censored for death from either CRC, another cancer, or another cause

For long-term survivors who were <65 years old at diagnosis, 50/62 (81 %) were alive at the end of follow-up, and 10 out of 12 deaths were related to CRC in this group. In patients who were >65 years old at diagnosis, 63 % were alive at the end of follow-up, and only 4 of 21 deaths were related to CRC.

The difference in the cancer-specific mortality was not significant. Altogether (Table 2), 12 men and only 2 women died of CRC after 5 years of systematic surveillance. In the univariate analysis, only female gender (*P* = 0.041) and TNM stage (*P* = 0.002) predicted cancer-specific survival after 5 years (Table 2).

**Fig. 3:**
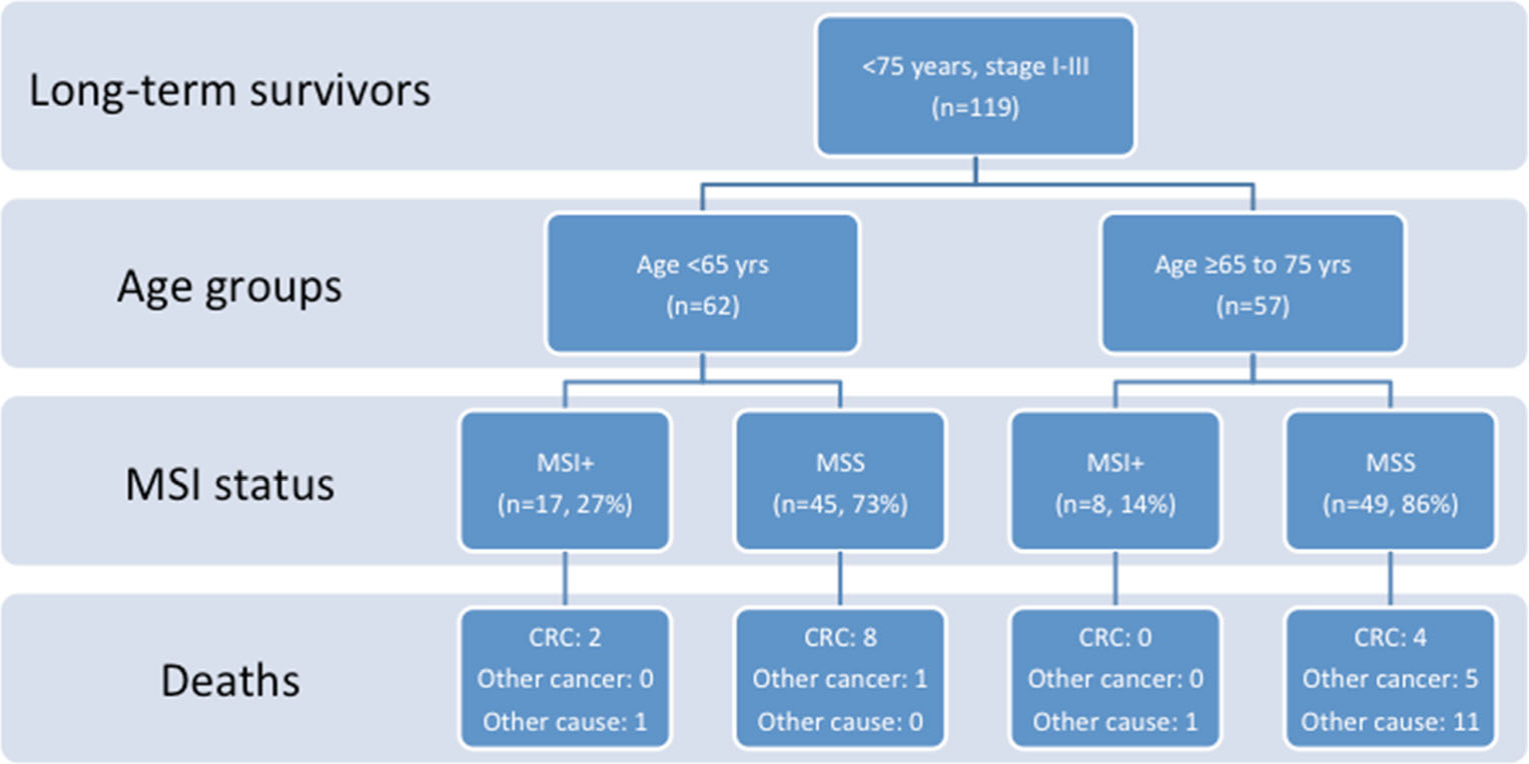
Flowchart demonstrating the distribution of long-term survivors who completed the 5-year surveillance programme (suggested as a supplementary figure only if needed due to space restrictions). Distribution (*n* = 119) according to the age groups, MSI status and cause of death is depicted. *MSI*+ microsatellite instability, *MSS* microsatellite stability, *yrs* years, and *CRC* colorectal cancer-related death

Microsatellite instability (MSI) was detected in the tumours of 25 (21 %) patients. Within the node-negative group, 24 % of tumours exhibited MSI, whereas only 11 % of tumours exhibited MSI in the node-positive group. In the younger patient group (<65 years old), more patients had MSI tumours than in the older group (>65 years), with rates of 27 % (17/62) and 14 % (8/57), respectively (Fig. 3). For patients who were alive after 5 years of follow-up, 14 died of CRC, and 10 were younger than 65 years. Only 2 of those younger than 65 years old who died of CRC exhibited MSI in their primary tumours.

## Discussion

This long-term study of patients who survived beyond 5 years after systematic surveillance demonstrated an overall good prognosis for node-negative disease. However, 12 % of patients died from CRC, and the numbers were equally distributed between node-negative and node-positive cancer cases. Notably, the majority of CRC-related deaths occurred in patients who were <65 years old at diagnosis, possibly indicating that surveillance is warranted for a longer time period for certain high-risk patients and that the identification of such risk factors is useful for tailored follow-up.

One immediate perceived risk in the youngest survivors would be that of an (undiagnosed) inherited cancer syndrome, such as hereditary non-polyposis colorectal cancer (HNPCC). However, based on MSI analysis, only 2 out of the 10 CRC-related deaths in patients <65 years old were associated with MSI tumours, and the presence of HNPCC can therefore not explain the results. One explanation is that younger patients likely harbour a different, aggressive cancer biology. Alternatively, younger patients may exhibit more senescent or viable tumour cells that may recur over time or may exhibit an indolent pattern of tumour relapse as observed in some other cancer forms (i.e. breast cancer), thereby conferring a higher risk of cancer-related death on long-term follow-up. Our findings replicate, to some extent, the previous finding that younger patients (aged 15–44 years old) with stage III disease had worse long-term survival after 5 years of follow-up compared with the other age groups [21]. Additionally, patients with young-onset (non-hereditary) CRC exhibit a different genetic profile than older patients [22]. A further explanation may be that younger patients have a longer expected survival than the older patients and therefore higher cumulative exposure to factors that may have caused CRC in the first place (the “field defect” in the colon), increasing their risk for recurrence or new cancer. Further investigation into the biology of the persistent cancer risk is warranted.

Long-term survival for patients with CRC is excellent beyond the 5 years of follow-up. Up to 5 years, most deaths are related to CRC, but from 5 to 15 years, the majority of deaths are related to other cancers or causes, especially in the group older than 65 years of age. Cancer-related deaths are most frequent in the group that is younger than 65 years, but the majority of patients experienced recurrence before reaching the end of the 5-year survival. In this study, age did not predict disease-free survival, although only 16 % (2/12) of deaths in patients younger than 65 years old were caused by other diseases, which is in contrast with 81 % (17/21) of non-CRC-related deaths in patients who are ≥65 years old. The prognostic influence of age on survival after 5 years is largely reduced in several studies [21, 23–25]. Additionally, patients who were >75 years old were not included in the current study; we excluded the group of elderly and frail patients who may die from various comorbid diseases.

Notably, management and therapy may have changed since the commencement of patient enrolment in this study. A small sample size is also a limitation. However, to investigate a long-term follow-up, a change in management over time is an inherent problem. The small sample size is in part due to the selected population; we chose only patients with stage I–III, a defined curative resection, age <75 years and who had entered a surveillance programme. This procedure selects for younger, healthier patients with less disease burden and for which the overall survival is expected to be very good. This study reveals that there is still an inherent risk of cancer death in these patients, and an investigation into the nature and biology of this finding is warranted. Future studies should also aim for a larger sample size.

In the present study, the TNM stage predicted cancer-specific survival. This result is in contrast with a larger Dutch study from 2007 [24] in which the TNM stage had no predictive value for patients who survived 5 years after diagnosis. Several other studies demonstrated that as time goes by, stage loses its prognostic significance when investigated for duration cut-offs at 5 or 8 years of follow-up after diagnosis [21, 24]. This effect may be because the cancer-specific death rate declines as the years progress, whereas the rate is the most prominent for stage III during the early stages of follow-up (the first 3 years after surgery) [26, 27].

In addition to stage, female gender predicted better cancer-specific survival after 5 years in the current study. This may be explained by the larger mortality for females within the first 5 years after diagnosis as well as the higher percentage of males compared with females who were diagnosed with TNM stage III. According to the national figures from the Cancer Registry of Norway, females have better short- and long-term colon cancer survival than males [28]. The contribution of gender to the prognosis requires further investigation.

The current study points to a still-present risk factor for cancer-related events in node-negative CRC, although survival beyond 5 years remains very good. The strength of the current study is the population-based approach, wherein we evaluated a well-defined cohort and performed a complete follow-up for all causes of death. However, some limitations are worth noting. The number of patients was limited in this cohort study, which may have obscured any true statistically significant results between groups because the sample size per group was small. Additionally, we cannot rule out other familiar or hereditary genetic risk syndromes because the only molecular test run for this cohort was the MSI analysis. However, no hereditary syndromes were reported in any of the patients, and this shortcoming applies to most other long-term follow-up studies where genetic testing is not a routine component of clinical care. Additionally, all patients were relatively young (<75 years old) at diagnosis; therefore, the cancer-specific outcome was not obscured by high age-related mortality from other causes. With the increasing indications for secondary metastatic surgery for both hepatic and pulmonary metastases as well as the improved outcomes from adjuvant chemotherapy, the identification of the patients who are at risk for recurrent disease has become increasingly important to tailoring the best surveillance strategy to the patients’ needs.

## Conclusions

Long-term survival beyond the 5-year surveillance period for stage I–III CRC is very good. Notably, cancer-related deaths are encountered in one-third of patients. Cancer-related deaths occur most frequently in patients who are <65 years old at diagnosis. This finding may warrant further investigation into long-term survival beyond the usual 5-year surveillance in patients who are young at their primary diagnosis.

## Abbreviations

OS: Overall survival
CSS: Cancer-specific survival
CRC: Colorectal cancer
ASA: American Society of Anesthesiologists score
MSI: Microsatellite instability
CEA: Carcinoembryonic antigen
TNM: Tumour-node-metastasis stage
NGICG: Norwegian Gastrointestinal Cancer Group
